# Supplementary material for: In situ combinatorial synthesis of degradable branched lipidoids for systemic delivery of mRNA therapeutics and gene editors
Source: Nat Commun. 2024 Feb 26;15:1762. doi: 10.1038/s41467-024-45537-z (PMC10897129; doi:10.1038/s41467-024-45537-z)
Supplement: Supplementary file 1 — Supplementary Information [file 41467_2024_45537_MOESM1_ESM.pdf]

Supplementary Information for

In situ combinatorial synthesis of degradable branched lipidoids for systemic delivery of mRNA therapeutics and gene editors

Xuexiang Han[1†,#], Junchao Xu[1†], Ying Xu[2], Mohamad-Gabriel Alameh[3,4], Lulu Xue[1], Ningqiang Gong[1], Rakan El-Mayta[3], Rohan Palanki[1], Claude C. Warzecha[5], Gan Zhao[6], Andrew E. Vaughan[6], James M. Wilson[5], Drew Weissman[3,4], Michael J. Mitchell[1,4,7,8,9,10]*

[1]Department of Bioengineering, University of Pennsylvania, Philadelphia, PA 19104, USA
[2]Department of Chemistry, Case Western Reserve University, Cleveland, OH 44106, USA
[3]Department of Medicine, University of Pennsylvania, Philadelphia, PA 19104, USA
[4]Penn Institute for RNA Innovation, Perelman School of Medicine, University of Pennsylvania, Philadelphia, PA 19104, USA
[5]Gene Therapy Program, Perelman School of Medicine, University of Pennsylvania, Philadelphia, PA 19104, USA
[6]Department of Biomedical Sciences, School of Veterinary Medicine, University of Pennsylvania, Philadelphia, PA 19104, USA
[7]Abramson Cancer Center, Perelman School of Medicine, University of Pennsylvania, Philadelphia, PA 19104, USA
[8]Institute for Immunology, Perelman School of Medicine, University of Pennsylvania, Philadelphia, PA 19104, USA
[9]Cardiovascular Institute, Perelman School of Medicine, University of Pennsylvania, Philadelphia, PA 19104, USA
[10]Institute for Regenerative Medicine, Perelman School of Medicine, University of Pennsylvania, Philadelphia, PA 19104, USA
[†]These authors contributed equally to this work.
[#]Present address: Key Laboratory of RNA Innovation, Science and Engineering, CAS Center for Excellence in Molecular Cell Science, Shanghai Institute of Biochemistry and Cell Biology, Chinese Academy of Sciences, University of Chinese Academy of Sciences, 320 Yue Yang Road, Shanghai, 200031, China
[*]Corresponding author: Michael J. Mitchell
Email: mjmitch@seas.upenn.edu

This PDF file includes:

Figures S1 to S19
Tables S1 to S5

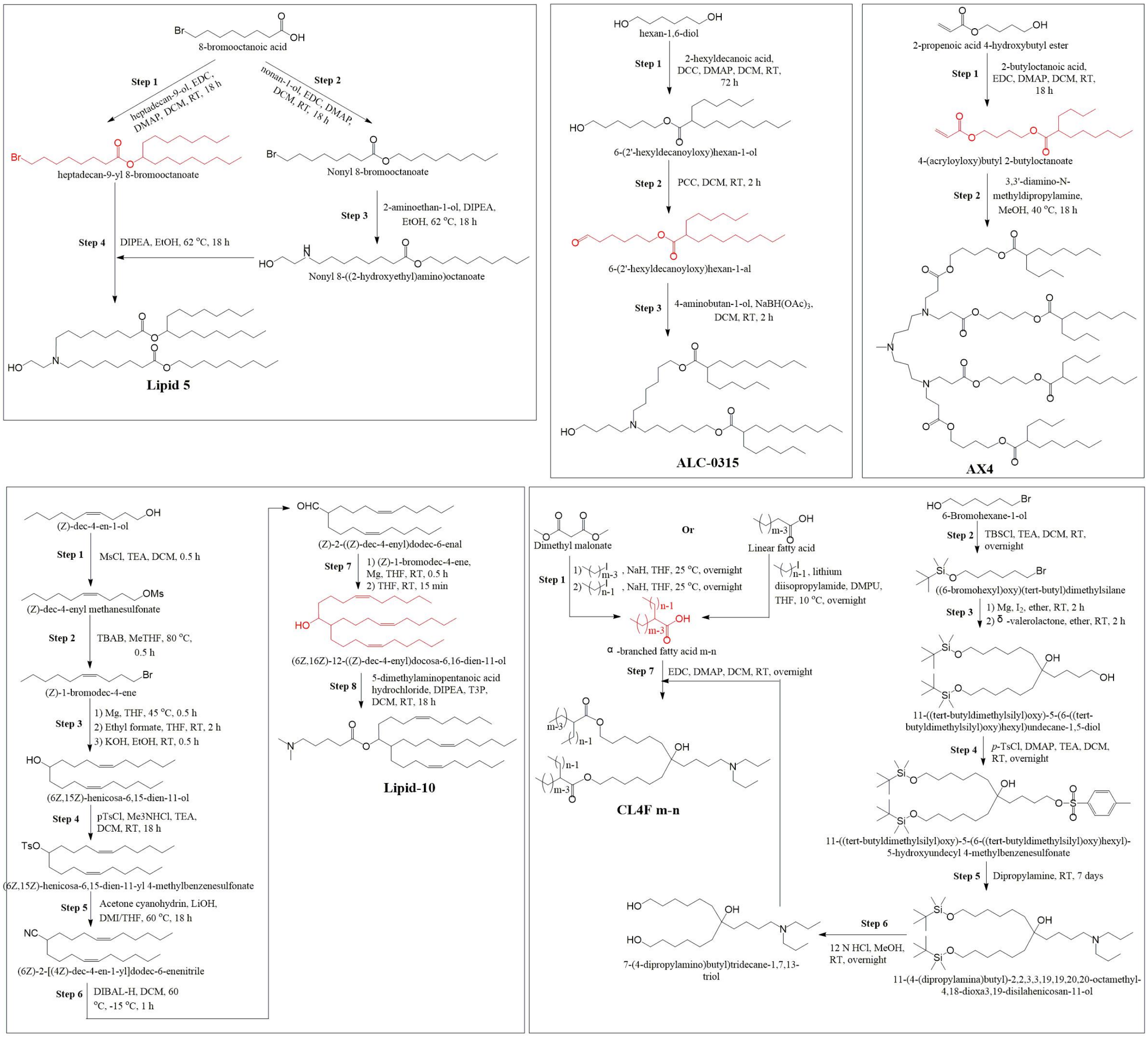

Figure S1. Summary of representative synthetic routes for degradable lipidoids with extended alkyl branches from the literature. The synthetic routes of Moderna's Lipid 5[1], Acuitas's ALC-0315[2], AX4[3], Genevant's Lipid-10[4] and CL4F m-n lipids[5] are shown. These lipidoids were synthesized based on two main steps: first, the preparation of a branched tail intermediate containing a functional group (highlighted in red); second, the connection of branched tail(s) to the headgroup. This method involves multiple synthetic steps and purifications with limited capacity (due to the lack of readily available branched intermediate) to generate a large library of degradable branched lipidoids.

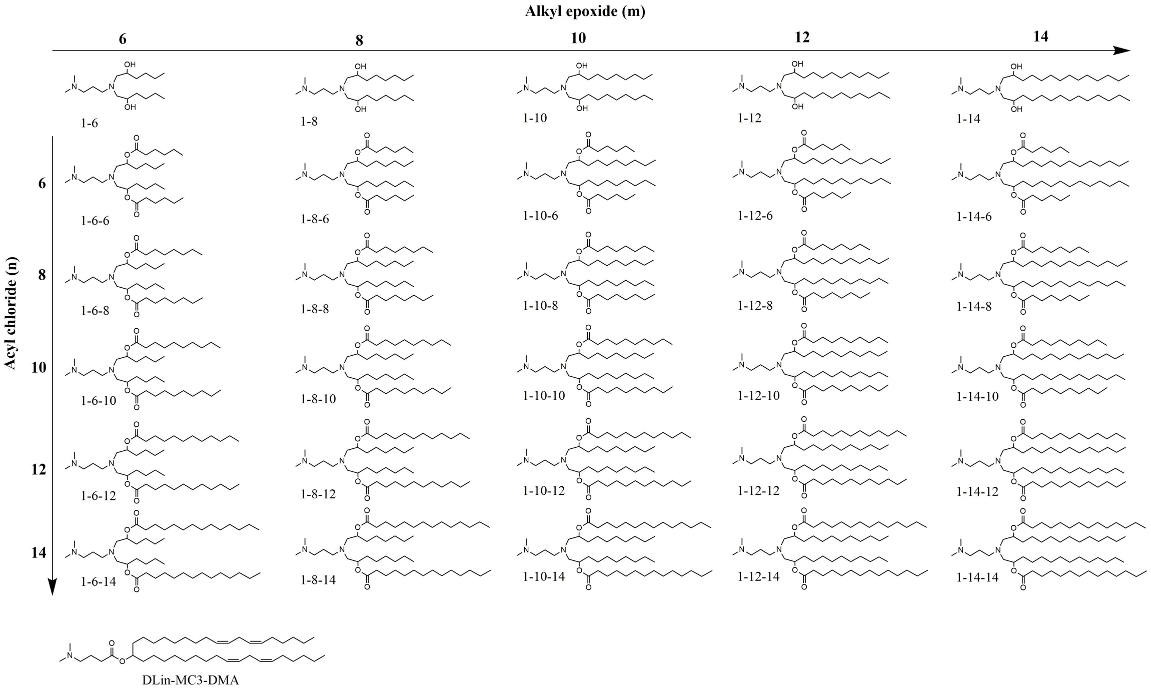

Figure S2. Chemical structures of DB-lipidoids in Library 1, aminoalcohol lipidoids and DLin-MC3-DMA (MC3).

a

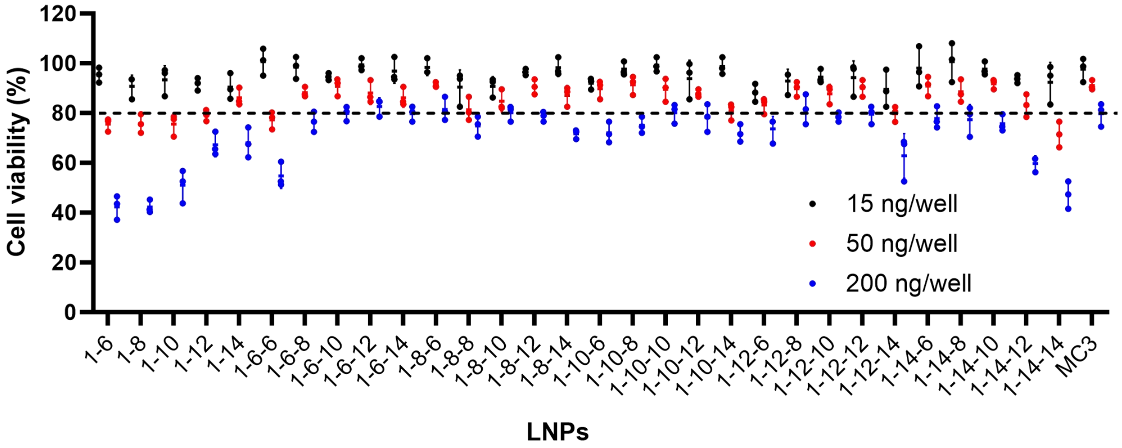

b

LNP	IC_{50} (ng/well)
1-6	148.9
1-8	148.3
1-10	>200
1-12	>200
1-14	>200
1-6-6	>200
1-6-8	>200
1-6-10	>200
1-6-12	>200
1-6-14	>200
1-8-6	>200
1-8-8	>200
1-8-10	>200
1-8-12	>200
1-8-14	>200
1-10-6	>200
1-10-8	>200
1-10-10	>200
1-10-12	>200
1-10-14	>200
1-12-6	>200
1-12-8	>200
1-12-10	>200
1-12-12	>200
1-12-14	>200
1-14-6	>200
1-14-8	>200
1-14-10	>200
1-14-12	>200
1-14-14	167.3
MC3	>200

Figure S3. Cell viability and IC_{50} of DB-LNPs in Library 1 and other LNPs. (a) Cell viability. HepG2 cells were treated with LNPs at 15 ng mRNA/well (0.24 nM), 50 ng mRNA/well (0.8 nM) or 200 ng mRNA/well (3.2 nM) for 24 h. No obvious cytotoxicity was observed for all LNPs at a low dose (*i.e.*, 15 ng mRNA/well). The dashed line indicates 80% cell viability. Data are presented as mean ± SD (n = 3 biologically independent samples). (b) Half-maximal inhibitory concentration (IC_{50}). IC_{50} was determined by nonlinear regression of dose and cell viability using GraphPad Prism 8.0. Source data are provided as a Source Data file.

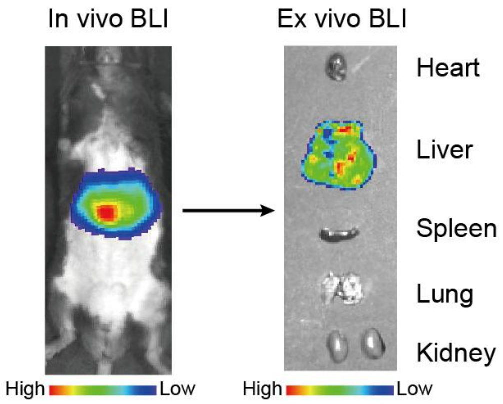

Figure S4. Representative results of *in vivo* and *ex vivo* bioluminescence imaging (BLI) during LNP screening. Mice were i.v. injected with mLuc-loaded DB-LNPs at an mRNA dose of 0.1 mg/kg. Images were taken at 4 h post-treatment. The signal mainly localized in the upper abdomen based on *in vivo* BLI, which was identified to come from the liver by *ex vivo* BLI.

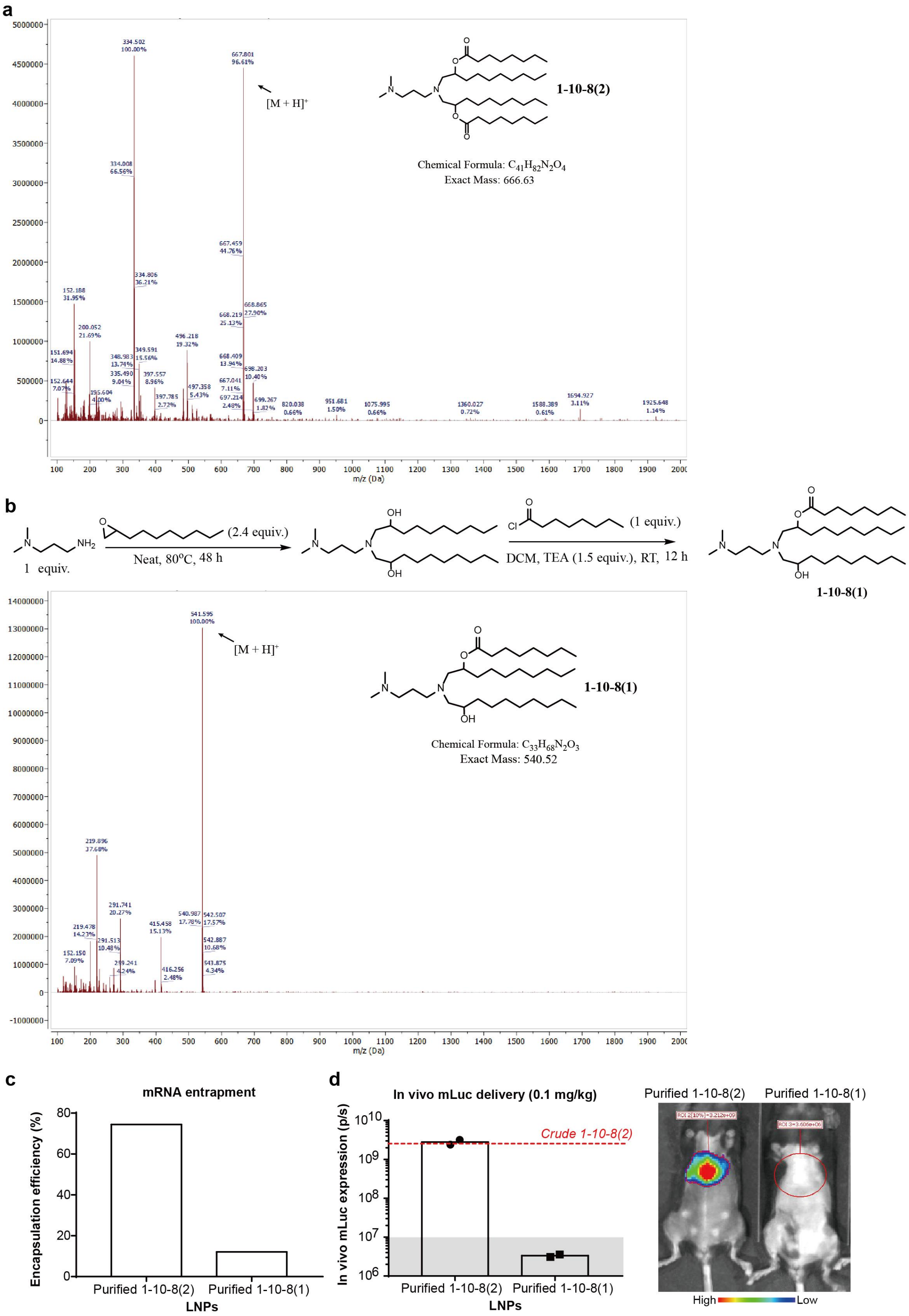

Figure S5. Comparison of purified 1-10-8(2) and 1-10-8(1). (a) Mass spectrum of 1-10-8(2). (b) Synthetic route and mass spectrum of 1-10-8(1). (c) mRNA encapsulation

efficiencies of 1-10-8(2) LNP and 1-10-8(1) LNP. (d) *In vivo* mLuc expression (n = 2 biologically independent samples). The grey shadow indicates background level. Lipidoids were purified using a CombiFlash NextGen 300+ chromatography system, and the desired fractions were collected and confirmed by mass spectrum. LNPs were formulated by pipette mixing. Source data are provided as a Source Data file.

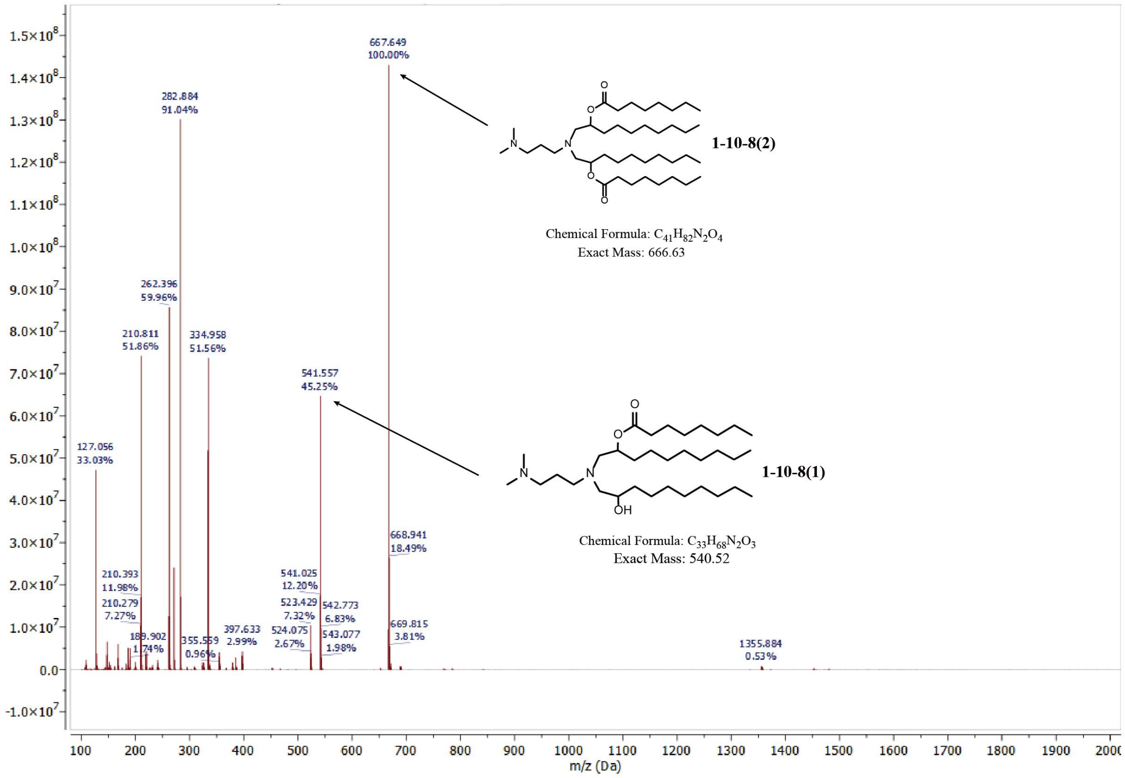

Figure S6. Mass spectrum of esterase-treated 1-10-8(2). 1-10-8(2) at 0.1 mg/mL was incubated with porcine liver esterase (100 U/mL) in PBS buffer at 37 °C for 1 h. The incubation solution was mixed with 10-fold volume of acetonitrile to terminate the esterase reaction, and the mixture was centrifuged at 10,000 rpm for 5 min. The supernatant was analyzed using mass spectrum. The presence of metabolite 1-10-8(1) was confirmed.

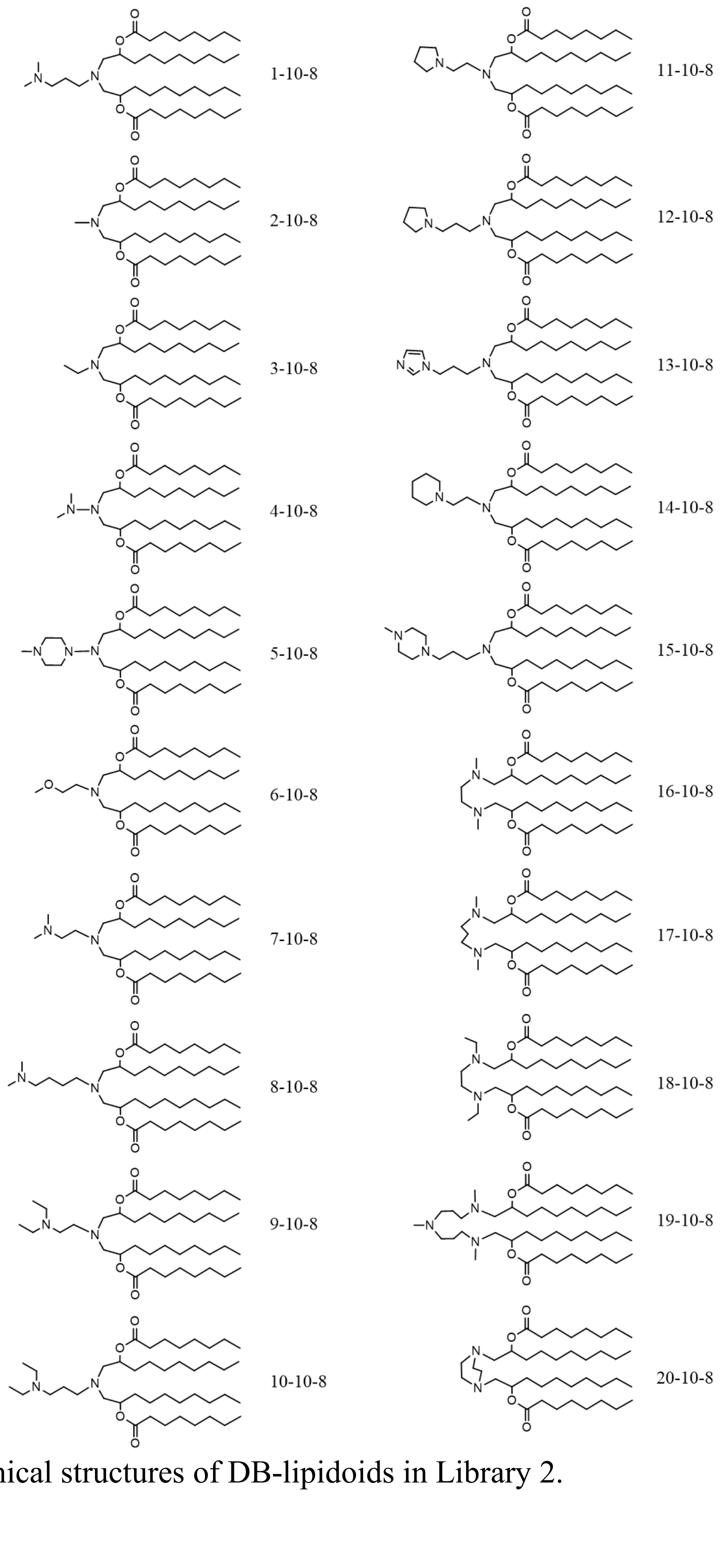

Figure S7. Chemical structures of DB-lipidoids in Library 2.

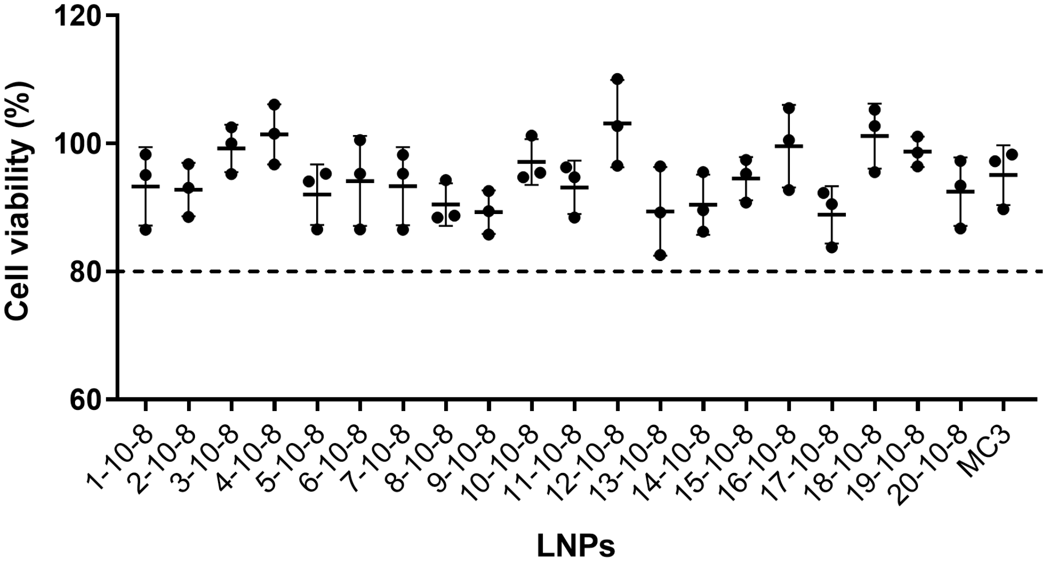

Figure S8. Cell viability of DB-LNPs in Library 2. HepG2 cells were treated with various LNPs at a dose of 15 ng mRNA/well (0.24 nM) for 24 h. No obvious cytotoxicity was induced by DB-LNPs. The dashed line indicates 80% cell viability. Data are presented as mean ± SD (n = 3 biologically independent samples). Source data are provided as a Source Data file.

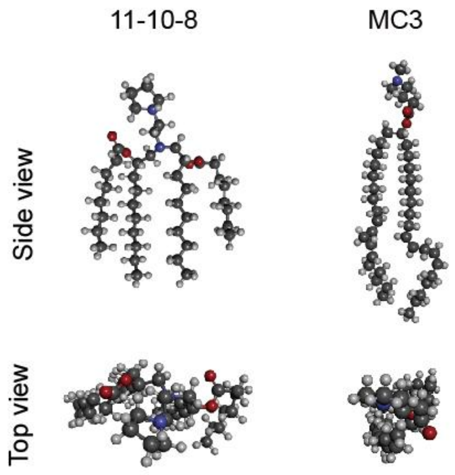

Packing parameters	11-10-8	MC3
V	1586.88	1664.42
A	35.34	34.99
L	11.02	22.55
P value	4.07	2.11

Figure S9. Structural illustration of 11-10-8 and MC3. Critical packing parameters were calculated based on molecular dynamics simulations. The dimensionless packing parameter P of a lipid molecule was calculated as $P = V/(AL)$ based on its Van der Waals molecule volume (V), cross section area of polar head (A) and average tail length (L).

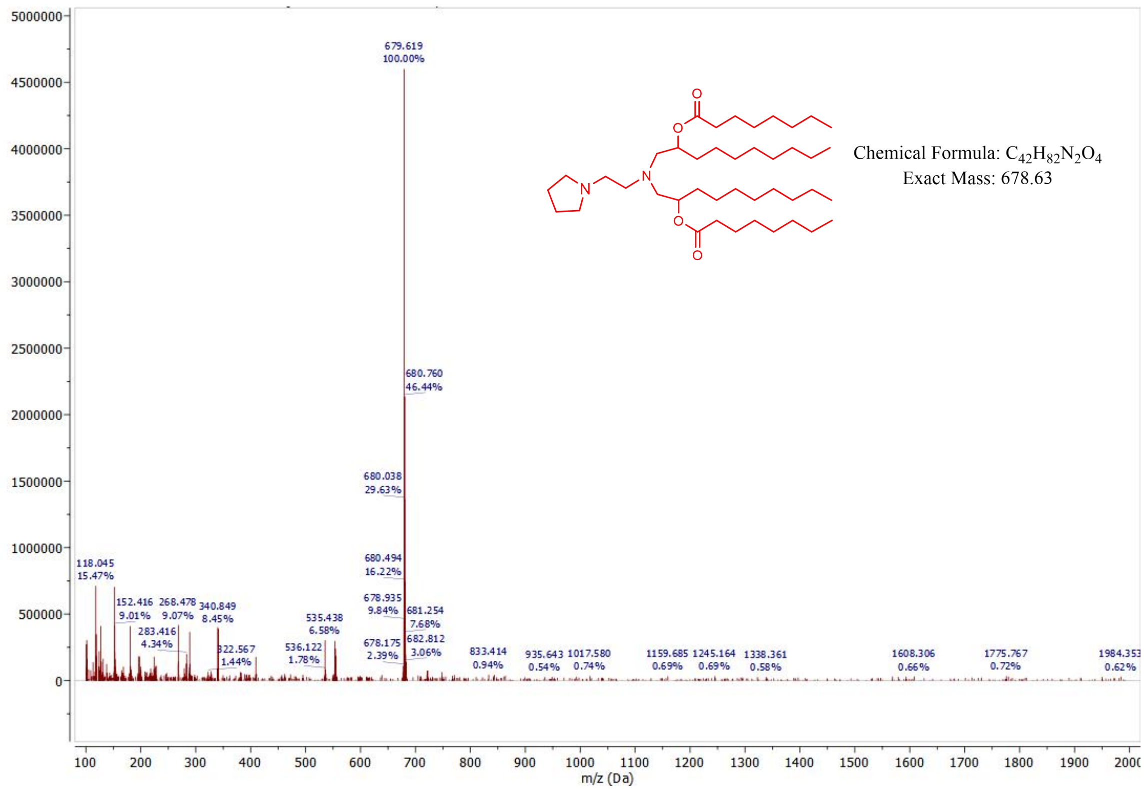

Figure S10. Mass spectrum of purified 11-10-8 DB-lipidoid.

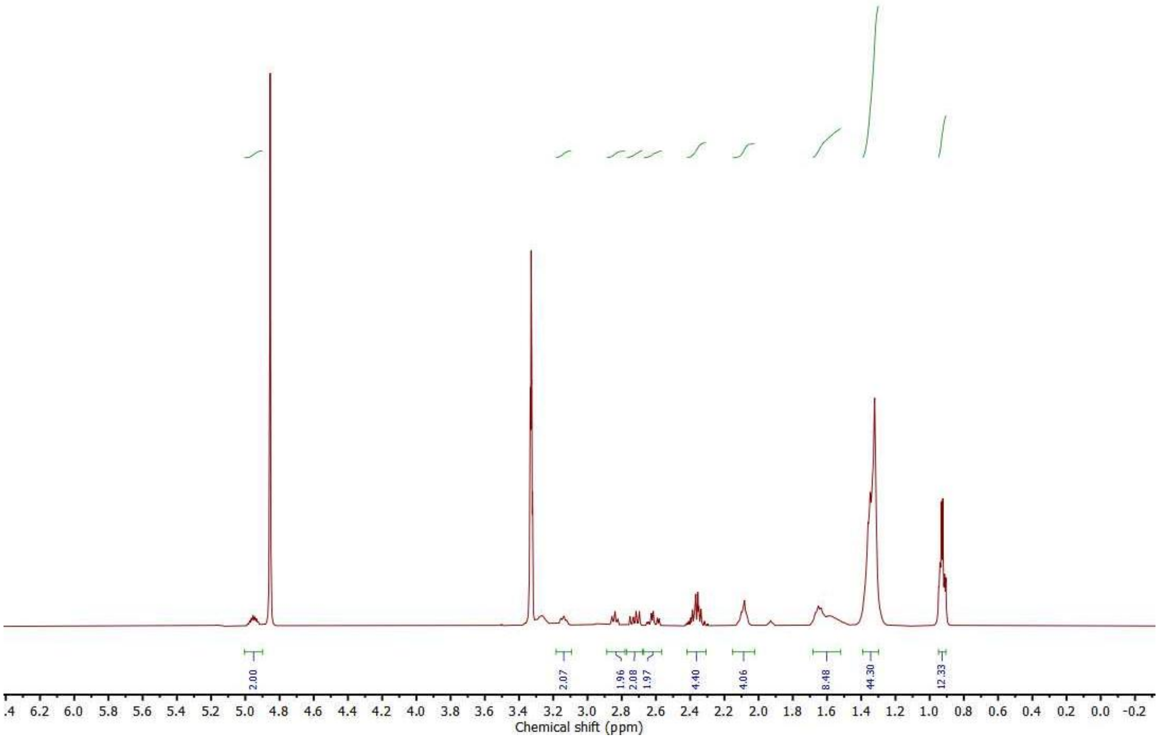

Figure S11. ^{1}H-NMR spectrum of purified 11-10-8 DB-lipidoid in MeOD.

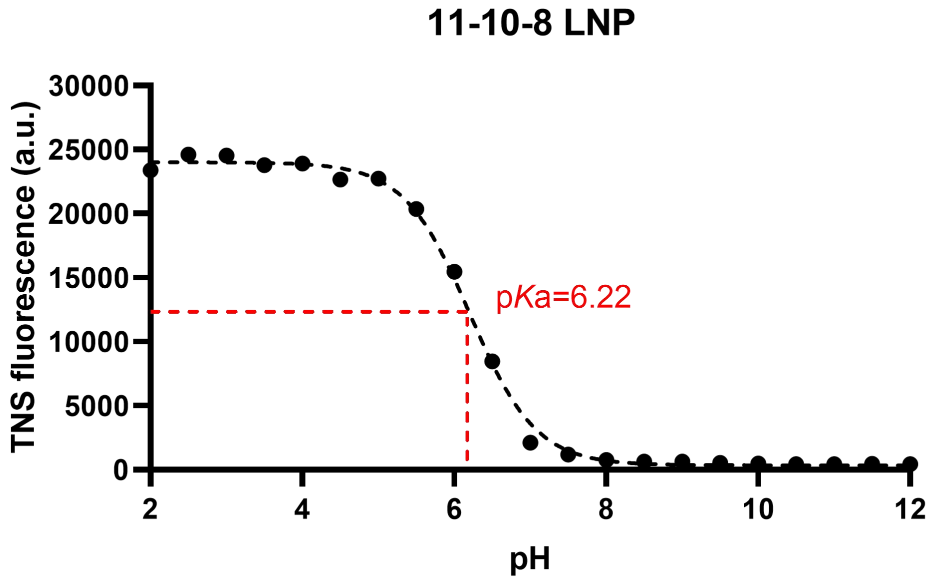

Figure S12. TNS assay was used to determine the apparent pK_a of 11-10-8 LNP. TNS fluorescence signal corresponds to ionization. pK_a is calculated as the pH corresponding to half of the maximum TNS fluorescence value. Source data are provided as a Source Data file.

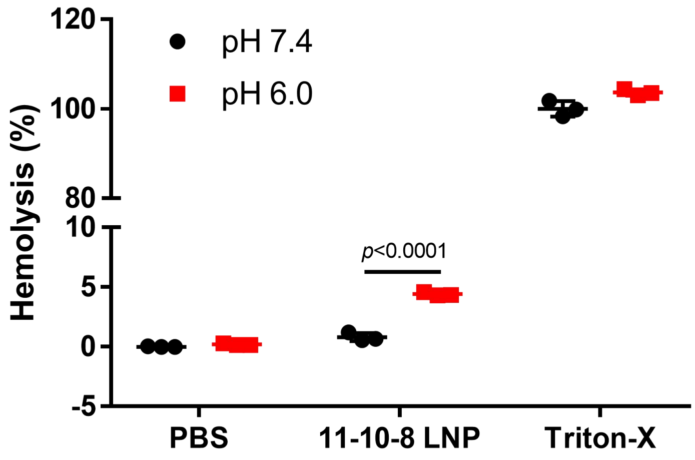

Figure S13. Hemolysis of 11-10-8 LNP at pH 7.4 or 6.0. RBCs were incubated with 11-10-8 LNP at an mRNA concentration of 3 μg/mL at 37 °C for 1 h. Positive and negative controls were carried out with 0.1% Triton-X and 1× PBS, respectively. Data are presented as mean ± SD (n = 3 biologically independent samples). Source data are provided as a Source Data file.

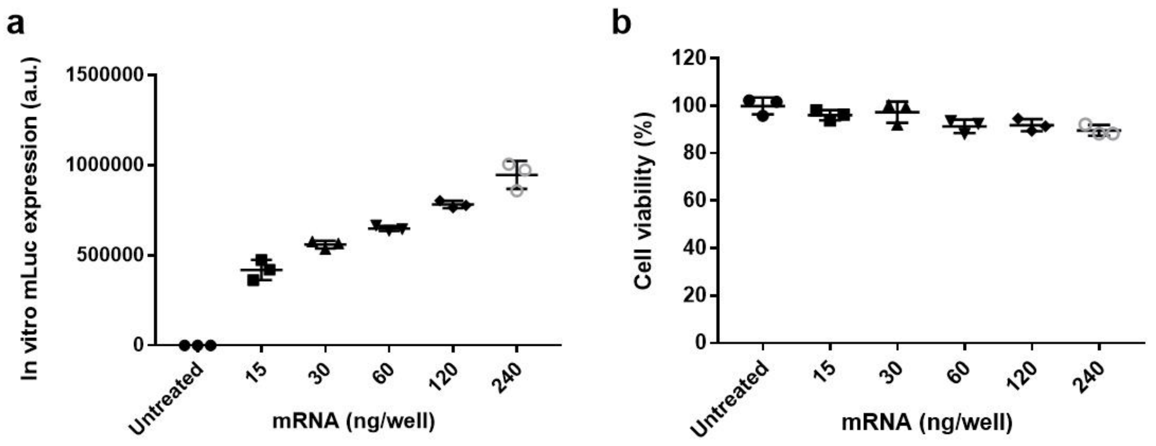

Figure S14. 11-10-8 LNP-mediated *in vitro* mLuc delivery and cytotoxicity. (a) *In vitro* mLuc expression. (b) Cell viability. HepG2 cells were treated with 11-10-8 LNP at indicated doses for 24 h. Data are presented as mean ± SD (n = 3 biologically independent samples). Source data are provided as a Source Data file.

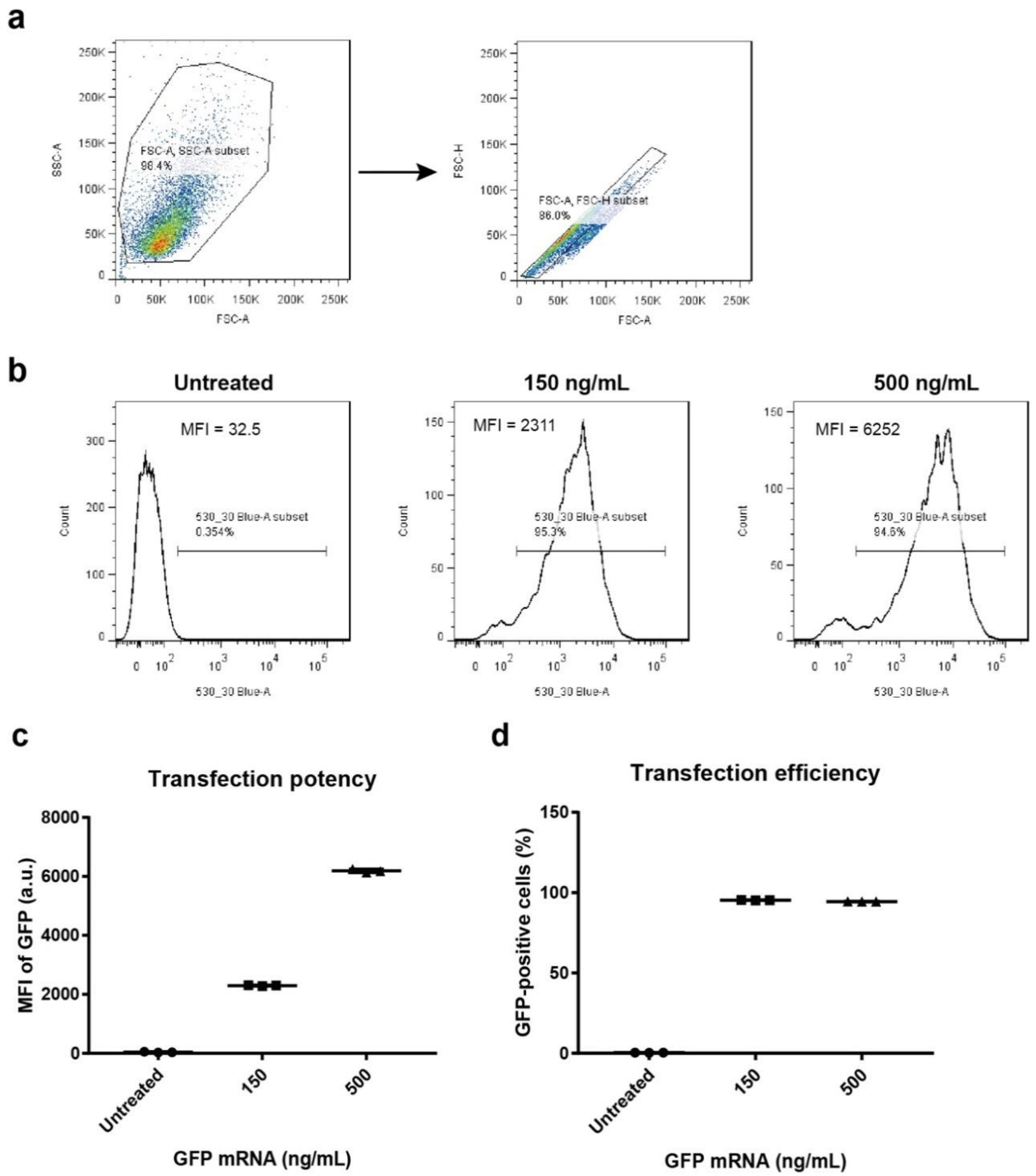

Figure S15. Flow cytometry analysis of GFP mRNA expression in HepG2 cells. (a) Gating strategy. (b) Representative results of GFP expression. (c) Mean fluorescence intensity (MFI) of cells. (d) Percentage of GFP-positive cells. HepG2 cells were treated with GFP mRNA-loaded 11-10-8 LNP at the indicated doses for 24 h. Data are presented as mean ± SD (n = 3 biologically independent samples). Source data are provided as a Source Data file.

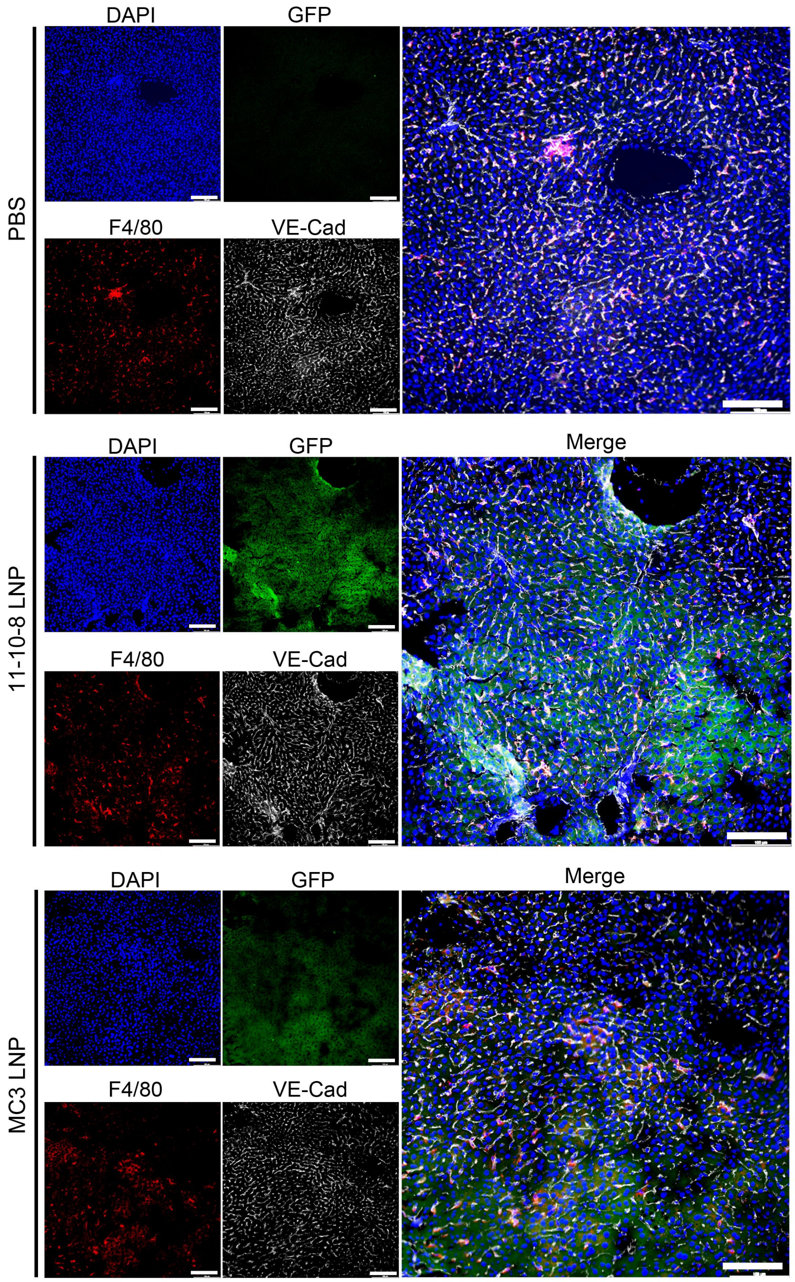

Figure S16. GFP expression in the liver at 4 h post-treatment of GFP mRNA-loaded LNPs. Mice were i.v. injected with GFP mRNA-loaded LNPs at an mRNA dose of 0.25mg/kg. Livers were collected and cryo-sectioned for immunofluorescence staining at 4 h post-treatment. Kupffer cells ($F4/80^{+}$) and liver sinusoidal endothelial cells ($VE\text{-}Cad^{+}$) were stained, respectively. Representative images from three independent experiments were shown. Scale bars: 100 µm.

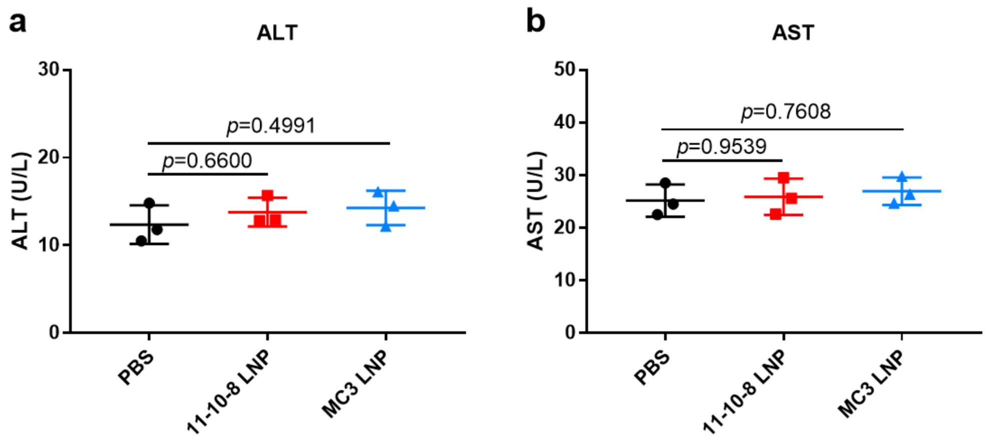

Figure S17. ALT and AST analysis. (a) Serum ALT (n = 3 biologically independent samples). (b) Serum AST (n = 3 biologically independent samples). Mice were i.v. injected with LNPs co-delivering Cas9 mRNA/TTR sgRNA (4:1, wt:wt) at a total RNA dose of 1 mg/kg. Serum was collected for ALT and AST analysis at 24 h post-treatment. Statistical significance was evaluated by a one-way ANOVA with Tukey's correction. Source data are provided as a Source Data file.

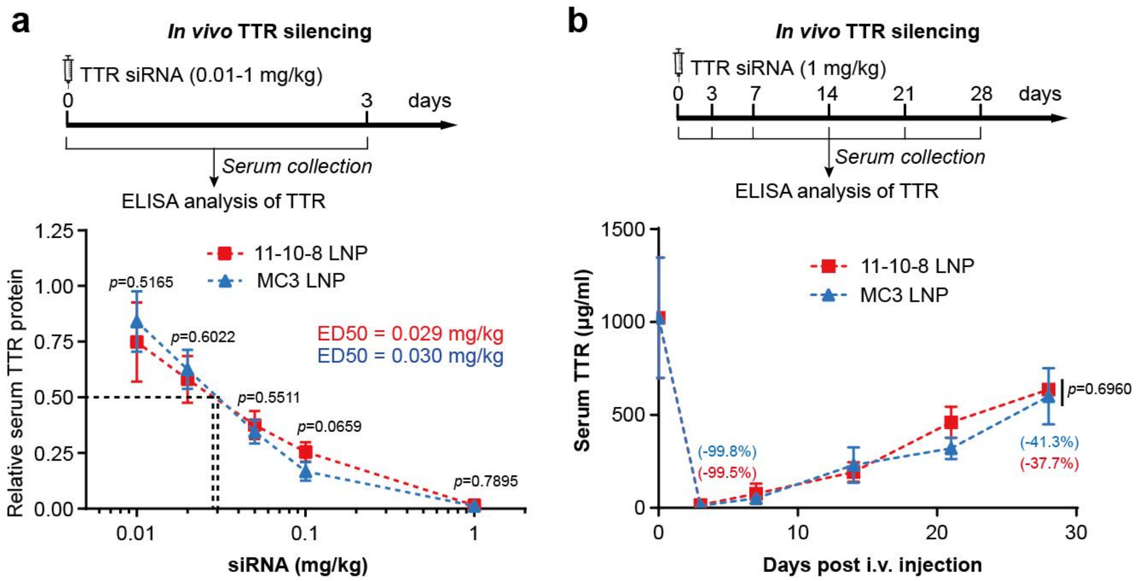

Figure S18. ***In vivo*** **LNP-mediated TTR siRNA delivery.** (a) Dose-dependent TTR silencing (n = 3 biologically independent samples). Mice were i.v. injected with TTR siRNA-loaded LNPs at different doses. Serum was collected on day 3 for ELISA analysis of serum TTR. Statistical significance was evaluated by an unpaired two-tailed Student's *t*-test. (b) Duration of TTR silencing (n = 3 biologically independent samples). Mice were i.v. injected with TTR siRNA-loaded LNPs at a dose of 1 mg/kg. Serum was collected at indicated time points for ELISA analysis of serum TTR. Statistical significance was evaluated by an unpaired two-tailed Student's *t*-test. Data are presented as mean ± SD. Source data are provided as a Source Data file.

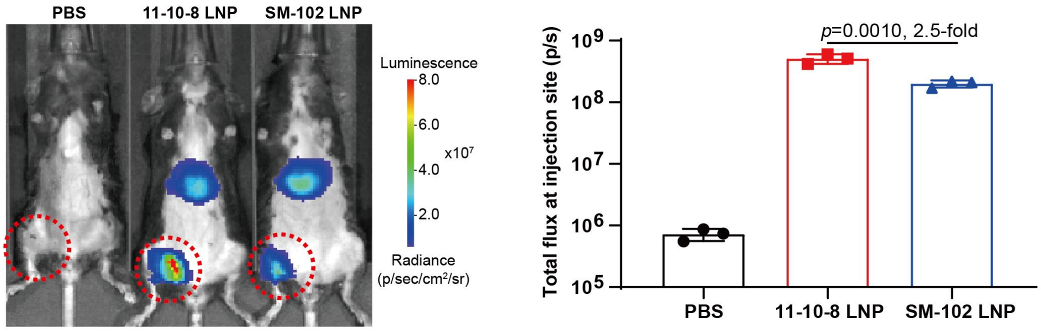

Figure S19. *In vivo* mLuc expression after i.m. injection of LNPs. Mice were i.m. injected with mLuc-loaded LNPs at an mRNA dose of 0.1 mg/kg. Images were taken at 4 h post-treatment. Data are presented as mean ± SD (n = 3 biologically independent samples). Statistical significance was evaluated by a one-way ANOVA with Tukey's correction. Source data are provided as a Source Data file.

Table S1. A summary of the observed m/z ratios in the mass spectra of DB-lipidoids.

Library	DB-lipidoid	Calculated	Observed
Library 1	1-6-6	498.44	499.50
	1-6-8	554.50	555.62
	1-6-10	610.56	611.56
	1-6-12	666.63	667.73
	1-6-14	722.69	723.71
	1-8-6	554.50	555.46
	1-8-8	610.56	611.46
	1-8-10	666.63	667.73
	1-8-12	722.69	723.59
	1-8-14	778.75	779.85
	1-10-6	610.56	611.46
	1-10-8	666.63	667.80
	1-10-10	722.69	723.79
	1-10-12	778.75	779.67
	1-10-14	834.82	835.71
	1-12-6	666.63	667.73
	1-12-8	722.69	723.79
	1-12-10	778.75	779.85
	1-12-12	834.82	835.90
	1-12-14	890.88	891.92
	1-14-6	722.69	723.79
	1-14-8	778.75	779.75
	1-14-10	834.82	835.92
	1-14-12	890.88	891.82
	1-14-14	946.94	948.01
Library 2	1-10-8	666.63	667.80
	2-10-8	595.55	596.75
	3-10-8	609.57	610.58
	4-10-8	624.58	625.48
	5-10-8	679.62	680.74
	6-10-8	639.58	640.79
	7-10-8	652.61	653.62
	8-10-8	680.64	681.54
	9-10-8	680.64	681.76
	10-10-8	694.66	695.87
	11-10-8	678.63	679.62
	12-10-8	692.64	693.65
	13-10-8	689.61	690.51
	14-10-8	692.64	693.76
	15-10-8	721.67	722.88
	16-10-8	652.61	653.62
	17-10-8	666.63	667.53
	18-10-8	680.64	681.76
	19-10-8	737.70	738.91
	20-10-8	650.60	651.58
Others	11-6-12	678.63	679.61
	11-8-10	678.63	679.62
	11-12-6	678.63	679.61
	11-12-10	758.69	759.70

Table S2. Characterization of MC3 LNP, aminoalcohol lipidoid-formulated LNPs and DB-LNPs in Libarary 1.

LNP	Size (nm)	PDI	Zeta potential (mV)	Encapsulation efficiency (%)
1-6	134.3 ± 5.5	0.14	-3.4 ± 0.7	19.0 ± 0.9
1-8	147.3 ± 6.8	0.20	-2.3 ± 0.9	20.7 ± 2.0
1-10	139.7 ± 4.7	0.16	-2.7 ± 1.0	24.4 ± 1.0
1-12	157.7 ± 6.7	0.18	-2.1 ± 0.8	29.9 ± 4.0
1-14	148.7 ± 8.3	0.17	-1.6 ± 1.0	32.8 ± 3.2
1-6-6	135.3 ± 5.5	0.21	-0.7 ± 1.3	32.6 ± 2.2
1-6-8	156.7 ± 4.6	0.20	1.9 ± 1.1	42.7 ± 0.6
1-6-10	129.7 ± 4.5	0.14	-1.4 ± 1.1	62.6 ± 5.9
1-6-12	146.3 ± 14.0	0.16	1.4 ± 0.1	77.8 ± 1.8
1-6-14	157.0 ± 5.0	0.14	-2.2 ± 0.7	75.7 ± 1.3
1-8-6	195.0 ± 12.8	0.20	0.03 ± 0.4	39.1 ± 0.4
1-8-8	138.7 ± 9.6	0.17	-1.6 ± 1.1	62.4 ± 4.9
1-8-10	140.7 ± 4.0	0.20	-0.8 ± 1.0	70.1 ± 1.2
1-8-12	174.7 ± 7.5	0.20	-1.6 ± 1.9	82.1 ± 1.0
1-8-14	130.0 ± 4.4	0.19	-1.6 ± 0.3	76.7 ± 2.3
1-10-6	160.0 ± 6.9	0.17	-2.0 ± 0.3	59.6 ± 3.5
1-10-8	150.7 ± 4.2	0.16	-0.03 ± 0.6	78.5 ± 0.4
1-10-10	154.0 ± 8.2	0.18	1.3 ± 1.1	79.8 ± 1.4
1-10-12	131.7 ± 5.9	0.19	-2.6 ± 0.7	77.2 ± 1.8
1-10-14	154.3 ± 6.7	0.17	0.47 ± 0.6	61.8 ± 1.4
1-12-6	130.3 ± 4.7	0.15	-2.7 ± 0.7	79.8 ± 4.3
1-12-8	136.0 ± 5.6	0.15	-2.6 ± 1.1	81.4 ± 0.9
1-12-10	162.3 ± 7.6	0.20	-2.0 ± 1.5	76.1 ± 2.0
1-12-12	198.7 ± 4.7	0.19	-0.67 ± 1.1	73.2 ± 1.8
1-12-14	214.0 ± 21.7	0.22	-1.6 ± 0.8	50.9 ± 2.9
1-14-6	137.0 ± 3.5	0.20	-0.6 ± 1.9	78.8 ± 0.5
1-14-8	150.3 ± 3.5	0.21	1.1 ± 0.8	72.5 ± 2.1
1-14-10	134.7 ± 4.2	0.18	-0.33 ± 0.9	76.6 ± 1.6
1-14-12	227.3 ± 23.5	0.22	1.5 ± 1.0	58.6 ± 4.9
1-14-14	203.7 ± 10.2	0.23	0.13 ± 0.3	42.7 ± 0.8
MC3	145.8 ± 8.1	0.17	-1.4 ± 0.3	73.5 ± 2.1

LNPs were formulated by pipette mixing. The hydrodynamic size and PDI were obtained by dynamic light scattering (DLS) measurement in neutral PBS. The zeta potential of LNPs was determined by laser-Doppler electrophoresis. The mRNA encapsulation efficiency was determined using a modified Quant-iT RiboGreen RNA assay. Data are presented as mean ± SD (n = 3 biologically independent samples).

Table S3. LNP formulation tested and their sources.

Formulation	Recipe	Molar ratio	Weight ratio	Source
F1	11-10-8*/DOPE/Chol/DMG-PEG	36.8:21:40.4:1.8	16:10:10:3	/
F2	11-10-8/DOPE/Chol/DMG-PEG	36.8:21:40.4:1.8	16:10:10:3	/
F3	11-10-8/DOPE/Chol/DMG-PEG	30.6:17.5:50.4:1.5	16:10:15:3	In house
F4	11-10-8/DOPE/Chol/DMG-PEG	35:16:46.5:2.5	16:8:12:4.5	Ref.[6]
F5	11-10-8/DOPE/Chol/DMG-PEG	40:10:48.5:1.5	16:4.4:11:2.4	Ref.[7]
F6	11-10-8/DSPC/Chol/DMG-PEG	50:10:38.5:1.5	16:3.7:7:1.9	Ref.[8]
MC3 LNP	MC3/DSPC/Chol/DMG-PEG	50:10:38.5:1.5	16:3.7:7:1.9	

11-10-8* indicates crude 11-10-8. The weight ratio of lipidoid:RNA was kept at 10:1 during LNP formulation.

Table S4. Characterization of benchmark LNPs formulated by microfluidic mixing.

LNP	Molar ratio	Size (nm)	PDI	Zeta potential (mV)	Encapsulation efficiency (%)
MC3 LNP	MC3/DSPC/Chol/DMG-PEG = 50:10:38.5:1.5	64.5 ± 3.7	0.063	-1.3 ± 0.3	92.7 ± 1.3
SM-102 LNP	SM-102/DSPC/Chol/DMG-PEG = 50:10:38.5:1.5	70.1 ± 1.5	0.076	-0.5 ± 0.1	95.6 ± 2.4

The hydrodynamic size and PDI were obtained by dynamic light scattering (DLS) measurement in neutral PBS. The zeta potential of LNPs was determined by laser-Doppler electrophoresis. The mRNA encapsulation efficiency was determined using a modified Quant-iT RiboGreen RNA assay. Data are presented as mean ± SD (n = 3 biologically independent samples).

Table S5. Innate immune responses after LNP treatment (n = 3 biologically independent samples). The results of cytokine concentrations (in pg/mL) are presented below.

LNPs	Mouse No.	IFN-γ	CXCL1	TNF-α	CCL2	IL-12p70	CCL5	IL-1β	CXCL10	GM-CSF	IL-10	IFN-β	IFN-α	IL-6
Control	#1	<0.86273	0.59578	<0.49209	<2.56921	<0.09701	<0.39291	0.27692	<1.84321	<0.36468	<0.44062	<0.41327	<0.38091	<0.29444
	#2	<0.86273	0.61765	<0.49209	<2.56921	<0.09701	<0.39291	<0.27548	<1.84321	<0.36468	0.677674	<0.41327	<0.38091	<0.29444
	#3	<0.86273	0.83858	<0.49209	<2.56921	0.10067	<0.39291	<0.27548	1.96991	<0.36468	0.484852	<0.41327	<0.38091	<0.29444
11-10-8 LNP	#4	<0.86273	0.71050	<0.49209	<2.56921	<0.09701	0.54404	<0.27548	2.79788	<0.36468	0.613821	<0.41327	0.42651	<0.29444
	#5	<0.86273	0.62192	<0.49209	<2.56921	0.11071	0.43782	0.28975	3.42005	<0.36468	0.524590	<0.41327	0.38942	0.35976
	#6	<0.86273	0.60464	<0.49209	<2.56921	<0.09701	<0.39291	<0.27548	3.76349	<0.36468	0.524590	<0.41327	0.50840	<0.29444
MC3 LNP	#7	<0.86273	0.77333	<0.49209	<2.56921	<0.09701	0.42437	<0.27548	5.56919	<0.36468	0.543476	<0.41327	0.54182	0.76111
	#8	<0.86273	1.01819	<0.49209	<2.56921	<0.09701	0.71032	<0.27548	6.42526	<0.36468	0.579595	<0.41327	0.70700	<0.29444
	#9	<0.86273	0.64890	<0.49209	<2.56921	<0.09701	0.67270	<0.27548	2.97137	<0.36468	<0.44062	<0.41327	0.54730	<0.29444

“<” indicates the cytokine concentration is lower than the minimum detectable concentration. Cas9 mRNA/TTR sgRNA-loaded LNPs (1 mg/kg) were i.v. injected into mice and serum was collected at 24 h post treatment for cytokine analysis. All cytokines were either undetectable or at low levels (< 10 pg/mL) for both LNPs, which were comparable with the untreated control group.

References

1. S. Sabnis, E. S. Kumarasinghe, T. Salerno, C. Mihai, T. Ketova *et al.* A Novel Amino Lipid Series for mRNA Delivery: Improved Endosomal Escape and Sustained Pharmacology and Safety in Non-human Primates. *Mol. Ther.* **26**, 1509-1519 (2018).
2. M. J. Hope, B. Mui, J. C. P. Lin, C. J. Barbosa, T. D. Madden *et al.* (U.S. Patent No. 11,712,481, 2020).
3. K. Huang, N. Li, Y. Li, J. Zhu, Q. Fan *et al.* Delivery of circular mRNA via degradable lipid nanoparticles against SARS-CoV-2 delta variant. *bioRxiv*, 2022.2005. 2012.491597 (2022).
4. K. Lam, A. Leung, A. Martin, M. Wood, P. Schreiner *et al.* Unsaturated, Trialkyl Ionizable Lipids are Versatile Lipid-Nanoparticle Components for Therapeutic and Vaccine Applications. *Adv. Mater.* **35**, e2209624 (2023).
5. K. Hashiba, Y. Sato, M. Taguchi, S. Sakamoto, A. Otsu *et al.* Branching Ionizable Lipids Can Enhance the Stability, Fusogenicity, and Functional Delivery of mRNA. *Small Science* **3**, 2200071 (2023).
6. K. J. Kauffman, J. R. Dorkin, J. H. Yang, M. W. Heartlein, F. DeRosa *et al.* Optimization of Lipid Nanoparticle Formulations for mRNA Delivery in Vivo with Fractional Factorial and Definitive Screening Designs. *Nano Lett.* **15**, 7300-7306 (2015).
7. R. Zhang, R. El-Mayta, T. J. Murdoch, C. C. Warzecha, M. M. Billingsley *et al.* Helper lipid structure influences protein adsorption and delivery of lipid nanoparticles to spleen and liver. *Biomaterials science* **9**, 1449-1463 (2021).
8. M. Jayaraman, S. M. Ansell, B. L. Mui, Y. K. Tam, J. Chen *et al.* Maximizing the potency of siRNA lipid nanoparticles for hepatic gene silencing in vivo. *Angew. Chem. Int. Ed. Engl.* **51**, 8529-8533 (2012).
