# Supplementary material for: In situ combinatorial synthesis of degradable branched lipidoids for systemic delivery of mRNA therapeutics and gene editors
Source: Nat Commun. 2024 Feb 26;15:1762. doi: 10.1038/s41467-024-45537-z (PMC10897129; doi:10.1038/s41467-024-45537-z)
Supplement: Supplementary file 3 — Reporting Summary [file 41467_2024_45537_MOESM3_ESM.pdf]

nature portfolio

Corresponding author(s): Michael Mitchell

Last updated by author(s): Jan 12, 2024

Reporting Summary

Nature Portfolio wishes to improve the reproducibility of the work that we publish. This form provides structure for consistency and transparency in reporting. For further information on Nature Portfolio policies, see our Editorial Policies and the Editorial Policy Checklist.

Statistics

For all statistical analyses, confirm that the following items are present in the figure legend, table legend, main text, or Methods section.

n/a	Confirmed	
☐	☒	The exact sample size (*n*) for each experimental group/condition, given as a discrete number and unit of measurement
☐	☒	A statement on whether measurements were taken from distinct samples or whether the same sample was measured repeatedly
☐	☒	The statistical test(s) used AND whether they are one- or two-sided<br>*Only common tests should be described solely by name; describe more complex techniques in the Methods section.*
☒	☐	A description of all covariates tested
☐	☒	A description of any assumptions or corrections, such as tests of normality and adjustment for multiple comparisons
☐	☒	A full description of the statistical parameters including central tendency (e.g. means) or other basic estimates (e.g. regression coefficient) AND variation (e.g. standard deviation) or associated estimates of uncertainty (e.g. confidence intervals)
☐	☒	For null hypothesis testing, the test statistic (e.g. *F*, *t*, *r*) with confidence intervals, effect sizes, degrees of freedom and *P* value noted<br>*Give P values as exact values whenever suitable.*
☒	☐	For Bayesian analysis, information on the choice of priors and Markov chain Monte Carlo settings
☒	☐	For hierarchical and complex designs, identification of the appropriate level for tests and full reporting of outcomes
☒	☐	Estimates of effect sizes (e.g. Cohen's *d*, Pearson's *r*), indicating how they were calculated

Our web collection on statistics for biologists contains articles on many of the points above.

Software and code

Policy information about availability of computer code

Data collection	MestReNova 9.0, Flowjo V10, IVIS specturm, Zeiss LSM 710
Data analysis	Statistical analysis was performed on Graphpad Prism 8.0, flow cytometry data were analyzed on FlowJo software package (Flowjo V10), 1H NMR spectrum was analyzed by MestReNova x64, confocal images were analyzed by Zen 2011 software and molecular simulation was performed on BIOVIA Discovery Studio 2018.

For manuscripts utilizing custom algorithms or software that are central to the research but not yet described in published literature, software must be made available to editors and reviewers. We strongly encourage code deposition in a community repository (e.g. GitHub). See the Nature Portfolio guidelines for submitting code & software for further information.

Data

Policy information about availability of data

All manuscripts must include a data availability statement. This statement should provide the following information, where applicable:

- Accession codes, unique identifiers, or web links for publicly available datasets
- A description of any restrictions on data availability
- For clinical datasets or third party data, please ensure that the statement adheres to our policy

All data supporting the findings of this study are available within the article and its supplementary files. Any additional requests for information can be directed to, and will be fulfilled by, the corresponding author. DNA sequencing files can be accessed at the National Center for Biotechnology Information Sequence Read Archive (NCBI SRA) with accession code PRJNA1064156. Source data are provided with this paper.

Human research participants

Policy information about studies involving human research participants and Sex and Gender in Research.

Reporting on sex and gender	No human participants were involved in this study.
Population characteristics	No human participants were involved in this study.
Recruitment	No human participants were involved in this study.
Ethics oversight	No human participants were involved in this study.

Note that full information on the approval of the study protocol must also be provided in the manuscript.

Field-specific reporting

Please select the one below that is the best fit for your research. If you are not sure, read the appropriate sections before making your selection.

[x] Life sciences   [ ] Behavioural & social sciences   [ ] Ecological, evolutionary & environmental sciences

For a reference copy of the document with all sections, see nature.com/documents/nr-reporting-summary-flat.pdf

Life sciences study design

All studies must disclose on these points even when the disclosure is negative.

Sample size	No statistical methods were used to determine the sample size in the study. The sample sizes are clearly described in each figure legend. The sample size were determined by allowable error size, accuracy, resources, and need for statistical analysis (generally n>=3 throughout all the studies).
Data exclusions	No animals and/or data were excluded.
Replication	All experiments were repeated for at least three times and experimental findings were reproducible.
Randomization	For in vivo study, animal groups were randomized by body weight. For other experiments, all samples were randomly allocated into experimental groups, as there was no covariate in the study design.
Blinding	All the investigators were blinded to group allocation during data collection and analysis.

Reporting for specific materials, systems and methods

We require information from authors about some types of materials, experimental systems and methods used in many studies. Here, indicate whether each material, system or method listed is relevant to your study. If you are not sure if a list item applies to your research, read the appropriate section before selecting a response.

Materials & experimental systems

n/a	Involved in the study
	☒ Antibodies
	☒ Eukaryotic cell lines
☒	☐ Palaeontology and archaeology
	☒ Animals and other organisms
☒	☐ Clinical data
☒	☐ Dual use research of concern

Methods

n/a	Involved in the study
☒	☐ ChIP-seq
	☒ Flow cytometry
☒	☐ MRI-based neuroimaging

Antibodies

Antibodies used	Vascular endothelial cadherin (VE-Cad) antibody (1:200, AF1002, R&D), F4/80 antibody (1:200, #30325, CST), Alexa Fluor™ 647 conjugated donkey anti-goat IgG (H+L) cross-adsorbed secondary antibody (1:500, A-21447, Invitrogen), Alexa Fluor™ 568 conjugated donkey anti-rabbit IgG (H+L) cross-adsorbed secondary antibody (1:500, A10042, Invitrogen)
Validation	All antibodies were verified by the supplier and each lot has been quality tested. All the antibodies used are from commercial sources and have been validated by the vendors. Validation data are available on the manufacturer's website.

Eukaryotic cell lines

Policy information about cell lines and Sex and Gender in Research

Cell line source(s)	HepG2 cells were obtained from American Type Culture Collection (ATCC, #HB-8065). This cell line was tested negative for mycoplasma in University of Pennsylvania cell center.
Authentication	A short tandem repeat DNA profiling method was used to authenticate the cell lines and the results were compared with reference database.
Mycoplasma contamination	These cells were tested for mycoplasma contamination. No mycoplasma contamination was found.
Commonly misidentified lines (See ICLAC register)	No commonly misidentified cell lines were used.

Animals and other research organisms

Policy information about studies involving animals; ARRIVE guidelines recommended for reporting animal research, and Sex and Gender in Research

Laboratory animals	C57BL/6 female mice (6-8 weeks, 18-20 g) and C57BL/6 male mice (6-8 weeks, 22-24 g) were purchased from Jackson Laboratory. and housed in a specific-pathogen-free animal facility at ambient temperature (22 ± 2 °C), air humidity 40%–70% and 12-h dark/12-h light cycle.
Wild animals	No wild animal was used in this study.
Reporting on sex	Female mice were used for in most cases in this study, and sex should not affect the results. Male mice were used in high fat diet-induced obese model, since female hormones can affect the obesity progression resulting in variations.
Field-collected samples	The study did not involve samples collected from field.
Ethics oversight	All animal protocols were approved by the Institutional Animal Care and Use Committee (IACUC) of University of Pennsylvania (Protocol No. 806540), and animal procedures were performed in accordance with the Guidelines for Care and Use of Laboratory Animals at the University of Pennsylvania.

Note that full information on the approval of the study protocol must also be provided in the manuscript.

Flow Cytometry

Plots

Confirm that:

☒ The axis labels state the marker and fluorochrome used (e.g. CD4-FITC).

☒ The axis scales are clearly visible. Include numbers along axes only for bottom left plot of group (a 'group' is an analysis of identical markers).

☒ All plots are contour plots with outliers or pseudocolor plots.

☒ A numerical value for number of cells or percentage (with statistics) is provided.

Methodology

Sample preparation	Cells were trypsinized. Single-cell suspensions were obtained and and then analyzed by flow cytometry for GFP signal.
Instrument	BD LSR II
Software	FlowJo software package (Flowjo V10)
Cell population abundance	The absolute cells >= 10000 were analyzed for fluorescent intensity in the defined gate.
Gating strategy	In general, cells were first gated on FSC/SSC. Singlet cells were gated using FSC-H and FSC-A. GFP signal was analyzed.

☒ Tick this box to confirm that a figure exemplifying the gating strategy is provided in the Supplementary Information.
